# Editorial Note: NOXA-Induced Alterations in the Bax/Smac Axis Enhance Sensitivity of Ovarian Cancer Cells to Cisplatin

**DOI:** 10.1371/journal.pone.0354761

**Published:** 2026-07-28

**Authors:** The PLOS One Editors

The *PLOS One* Editors issue this Editorial Note for this article [1,2] because of concerns related to the peer review process. Readers are encouraged to approach the article [1] with careful consideration.
